# Complete Genome Sequence of a γ-Hexachlorocyclohexane-Degrading Bacterium, *Sphingobium* sp. Strain MI1205

**DOI:** 10.1128/genomeA.00246-16

**Published:** 2016-04-07

**Authors:** Michiro Tabata, Satoshi Ohhata, Yuki Nikawadori, Takuya Sato, Kouhei Kishida, Yoshiyuki Ohtsubo, Masataka Tsuda, Yuji Nagata

**Affiliations:** Graduate School of Life Sciences, Tohoku University, Sendai, Japan

## Abstract

Here, we report the complete genome sequence of a γ-hexachlorocyclohexane (γ-HCH)-degrading bacterium, *Sphingobium* sp. strain MI1205. The genome of MI1205 consists of two chromosomes and four plasmids with sizes of 33 to 292 kb. All the *lin* genes for γ-HCH metabolism are dispersed on the four plasmids.

## GENOME ANNOUNCEMENT

γ-Hexachlorocylohexane (γ-HCH; also called γ-BHC or lindane) is a chlorinated organic insecticide that has caused serious environmental problems due to its toxicity and long persistence in upland soils (1, 2). In this study, we determined the complete genome sequence of a γ-HCH-degrading bacterium, *Sphingobium* sp. strain MI1205, which was isolated from HCH-contaminated soil in Miyagi prefecture, Japan (3), and has been deposited in the Japan Collection of Microorganisms (JCM) under the accession number JCM 17233.

The MI1205 genome was sequenced using the 454 GS-FLX+ (Roche) system and the HiSeq 2000 (Illumina) mate-pair sequencing system, which was operated by Eurofins Genomics Inc., and 479,052 reads and 1,881,908 reads, respectively, were obtained. These reads were assembled using Newbler (Roche) to generate initial draft sequence data consisting of 16 scaffolds and 64 contigs. The finishing was facilitated using GenoFinisher and AceFileViewer (4). The complete genome sequence was annotated by the NCBI Prokaryotic Genome Automatic Annotation Pipeline (PGAAP), and the resulting annotation was subjected to manual curation using the annotation support tool of GenomeMatcher (5). By referencing annotation data obtained from the Microbial Genome Annotation Pipeline (http://www.migap.org), we corrected the start codon positions and added several genes that were missing in the PGAAP annotation.

The MI1205 genome consists of two circular chromosomes, Chr1 (3,351,250 bp, 62.3% G+C, 3,278 open reading frames [ORFs]) and Chr2 (567,154 bp, 62.4% G+C, 516 ORFs), and four circular plasmids, pMI1 (292,135 bp, 62.8% G+C, 297 ORFs), pMI2 (287,488 bp, 62.5% G+C, 320 ORFs), pMI3 (88,374 bp, 61.0% G+C, 102 ORFs), and pMI4 (32,974 bp, 63.0% G+C, 45 ORFs). Two chromosomes carry two rRNA operons and 50 tRNA genes. The MI1205-specified *linA*, *linB*, *linC*, and *linEb* genes, and *linRED* and *linGHIJ* clusters for conversion of γ-HCH to metabolites in tricarboxylic acid (TCA) cycle (6) are almost identical to those from an archetypal γ-HCH-degrading strain, *S. japonicum* UT26 (7), and are dispersed on the four plasmids, pMI1 (*linB*, *linC*, *linF*, *linEb,* and *linRED* and *linGHIJ* clusters), pMI2 (*linA* and *linRED* cluster, whose *linE* is missing by frameshift), pMI3 (two copies of *linB* and *linC*), and pMI4 (*linRED* cluster). Localization of all the *lin* genes for γ-HCH metabolism on multiple plasmids was also observed in another γ-HCH-degrading strain, *Sphingomonas* sp. MM-1 (8, 9), but distribution patterns of the *lin* genes are different to each other.

### Nucleotide sequence accession numbers.

Sequences with annotation information have been deposited in GenBank under accession numbers CP005188, CP005189, CP005190, CP005191, CP005192, and CP005193, for Chr1, Chr2, pMI1, pMI2, pMI3, and pMI4, respectively.

## References

[1] Lal R, Pandey G, Sharma P, Kumari K, Malhotra S, Pandey R, Raina V, Kohler HP, Holliger C, Jackson C, Oakeshott JG 2010 Biochemistry of microbial degradation of hexachlorocyclohexane and prospects for bioremediation. Microbiol Mol Biol Rev 74:58–80. doi:10.1128/MMBR.00029-09. 20197499 PMC2832351

[2] Vijgen J, Aliyeva G, Weber R 2013 The forum of the International HCH and Pesticides Association—a platform for international cooperation. Environ Sci Pollut Res 20:2081–2086. doi:10.1007/s11356-012-1170-z. 22961560

[3] Ito M, Prokop Z, Klvana M, Ohtsubo Y, Tsuda M, Damborský J, Nagata Y 2007 Degradation of β-hexachlorocyclohexane by haloalkane dehalogenase LinB from γ-hexachlorocyclohexane-utilizing bacterium *Sphingobium* sp. MI1205. Arch Microbiol 188:313–325. doi:10.1007/s00203-007-0251-8. 17516046

[4] Ohtsubo Y, Maruyama F, Mitsui H, Nagata Y, Tsuda M 2012 Complete genome sequence of *Acidovorax* sp. strain KKS102, a polychlorinated-biphenyl degrader. J Bacteriol 194:6970–6971. doi:10.1128/JB.01848-12. 23209225 PMC3510582

[5] Ohtsubo Y, Ikeda-Ohtsubo W, Nagata Y, Tsuda M 2008 GenomeMatcher: a graphical user interface for DNA sequence comparison. BMC Bioinformatics 9:376. doi:10.1186/1471-2105-9-376. 18793444 PMC2553346

[6] Nagata Y, Natsui S, Endo R, Ohtsubo Y, Ichikawa N, Ankai A, Oguchi A, Fukui S, Fujita N, Tsuda M 2011 Genomic organization and genomic structural rearrangements of *Sphingobium japonicum* UT26, an archetypal γ-hexachlorocyclohexane-degrading bacterium. Enzyme Microb Technol 49:499–508. doi:10.1016/j.enzmictec.2011.10.005. 22142724

[7] Nagata Y, Endo R, Ito M, Ohtsubo Y, Tsuda M 2007 Aerobic degradation of lindane (γ-hexachlorocyclohexane) in bacteria and its biochemical and molecular basis. Appl Microbiol Biotechnol 76:741–752. doi:10.1007/s00253-007-1066-x. 17634937

[8] Tabata M, Endo R, Ito M, Ohtsubo Y, Kumar A, Tsuda M, Nagata Y 2011 The *lin* genes for γ-hexachlorocyclohexane degradation in *Sphingomonas* sp. MM-1 proved to be dispersed across multiple plasmids. Biosci Biotechnol Biochem 75:466–472. doi:10.1271/bbb.100652. 21389627

[9] Tabata M, Ohtsubo Y, Ohhata S, Tsuda M, Nagata Y 2013 Complete genome sequence of the γ-hexachlorocyclohexane-degrading bacterium *Sphingomonas* sp. strain MM-1. Genome Announc 1(3):e00247-13. doi:10.1128/genomeA.00247-13. 23682148 PMC3656210

